# Intestinal Microbiota in Colorectal Cancer Surgery

**DOI:** 10.3390/cancers12103011

**Published:** 2020-10-16

**Authors:** Ioannis Koliarakis, Elias Athanasakis, Markos Sgantzos, Theodoros Mariolis-Sapsakos, Evangelos Xynos, Emmanuel Chrysos, John Souglakos, John Tsiaoussis

**Affiliations:** 1 Laboratory of Anatomy, School of Medicine, University of Crete, 70013 Heraklion, Greece; medp2011931@med.uoc.gr; 2 Department of General Surgery, University Hospital of Heraklion, 71110 Heraklion, Greece; eliasathanasakis@yahoo.gr (E.A.); atlscrete@med.uoc.gr (E.C.); 3 Laboratory of Anatomy, Faculty of Medicine, School of Health Sciences, University of Thessaly, 41334 Larissa, Greece; sgantzos@med.uth.gr; 4 Surgical Department, National and Kapodistrian University of Athens, Agioi Anargyroi General and Oncologic Hospital of Kifisia, 14564 Athens, Greece; tmariolis@nurs.uoa.gr; 5 Department of Surgery, Creta Interclinic Hospital of Heraklion, 71305 Heraklion, Greece; exynos@med.uoc.gr; 6 Laboratory of Translational Oncology, School of Medicine, University of Crete, 71003 Heraklion, Greece; souglak@uoc.gr

**Keywords:** colorectal cancer, surgery, intestinal microbiota, dysbiosis, bowel preparation, antibiotics, anastomotic leakage, surgical site infection, outcomes

## Abstract

**Simple Summary:**

The microbial communities of the intestine exist in a delicate balance with the human. Colorectal cancer is one of the most common gastrointestinal malignancies, and the microbiota seems to be related to it. The intestinal microbiota of patients after colorectal surgery is changed due to surgical stress and other perioperative factors. The occurrence of complications after colorectal cancer (CRC) surgery may depend on these bacterial shifts, which could also be associated with prognosis and survival in postoperative CRC patients.

**Abstract:**

The intestinal microbiota consists of numerous microbial species that collectively interact with the host, playing a crucial role in health and disease. Colorectal cancer is well-known to be related to dysbiotic alterations in intestinal microbiota. It is evident that the microbiota is significantly affected by colorectal surgery in combination with the various perioperative interventions, mainly mechanical bowel preparation and antibiotic prophylaxis. The altered postoperative composition of intestinal microbiota could lead to an enhanced virulence, proliferation of pathogens, and diminishment of beneficial microorganisms resulting in severe complications including anastomotic leakage and surgical site infections. Moreover, the intestinal microbiota could be utilized as a possible biomarker in predicting long-term outcomes after surgical CRC treatment. Understanding the underlying mechanisms of these interactions will further support the establishment of genomic mapping of intestinal microbiota in the management of patients undergoing CRC surgery.

## 1. Introduction

The human intestinal microbiota is a complex microbial ecosystem that maintains an intestinal homeostasis through constant synergistic interactions with the host [1]. However, compositional or functional alterations in microbiota could impair this balance, promoting the onset of diseases such as obesity, diabetes, inflammatory bowel disease, and gastrointestinal cancer [2]. Colorectal cancer (CRC) is the third most commonly diagnosed and the second cause of cancer-related mortality in both sexes [3]. Although many advances have been made regarding the improvement of CRC management, surgical resection remains the cornerstone of treatment. There is evidence supporting the crucial role of the intestinal microbiota in CRC tumorigenesis [4]; however, the impact of colorectal surgery on the microbiota is not fully elucidated.

Perioperative interventions, especially preoperative mechanical bowel preparation (MBP) and antibiotic prophylactic therapy, either orally or systemically, could cause significant shifts in microbiota composition and diversity [5]. Since intestinal microbiota plays an essential role in wound healing and immune modulation, the altered microbiota as a result of surgical stress and perioperative management may be associated with the occurrence of postoperative complications including anastomotic leakage (AL), as well as surgical site infections (SSI) [6]. Moreover, long-term oncological outcomes are mandatory in determining the selection of adjuvant chemotherapy after CRC surgery. Thus, it would be of great importance to analyze the intestinal microbiota as a possible mediator of postoperative complications and as a potential biomarker of CRC prognosis.

This review aims to summarize the available literature regarding the impact of perioperative interventions and surgical stress on the intestinal microbiota and its relation to postoperative complications and long-term outcomes following CRC surgery.

## 2. The Effect of Preoperative Mechanical Bowel Preparation on Intestinal Microbiota

Since intestinal microbiota is considered to be a potential source of microbial infection following CRC surgery, colorectal surgeons investigated ways to decrease the bacterial load. The solution came with the establishment of MBP, which reduces the fecal content usually through the use of an osmotic agent [7]. The most frequently administered agent is polyethylene glycol (PEG), a metabolically inert isotonic laxative, which offers a simple and convenient bowel cleansing effect without causing major side effects such as electrolytic imbalance [8].

The concept of MBP has long been a standard practice in colorectal surgery, as it offers numerous advantages such as allowing a more feasible colonic manipulation and facilitating the detection of small colorectal tumors via palpation [9]. However, many studies that focus on the clinical value of MBP have reported that MBP does not prevent any postoperative surgical complications related to infection, possibly aggravating these adverse effects [10,11]. Furthermore, the development of enhanced recovery after surgery (ERAS) has institutionalized guidelines for performing colorectal procedures in non-prepared colons without significantly affecting the surgical outcome [12]. Nevertheless, many colorectal surgeons in Europe and the United States tend to use MBP especially for left hemicolectomy, mainly with the exception of low anterior resection with coloanal anastomosis [13,14]. Thus, the preoperative use of MBP still remains a matter of debate regarding elective colorectal surgery.

The multi-dimensional cleansing effect of MBP implies major alterations in the intestinal microbiota during preoperative preparation, and to date, only a few studies have characterized the impact of MBP on luminal and mucosa-associated microbiota. Microbiome analysis via 16S rRNA sequencing in patients undergoing sigmoidoscopy revealed a significant reduction of diversity in luminal and mucosa-associated microbiota after MBP, with the alterations being more profound at the genus level [15]. However, another study using high-throughput sequencing (HTS) and denaturing gradient gel electrophoresis (DGGE) showed non-specific transient alterations in microbiota composition after MBP [16]. Jalanka et al. [17] reported that the overall microbial population was significantly diminished immediately following MBP, but it returned to baseline levels within 14 days. The microbiota composition was also altered, with a reduction in *Bacilli* and *Clostridium* cluster IV and increase in *Clostridium* cluster XIVa and *Proteobacteria*. The administration method also affected the changes in microbiota, with the single-dose MBP causing more prominent and long-lasting bacterial perturbations than the double-split method, notably increasing members of *Proteobacteria* and *Fusobacteria*. Contrary to previous studies, Drago et al. [18] demonstrated major alterations in intestinal microbiota following MBP persisting for as long as 30 days, especially reducing *Lactobacillaceae* and increasing the abundance of *Enterobacteriaceae* and *Streptococcaceae*. A recently published paper [19], in accordance with previous studies [17], showed decreased microbiota composition and significant alterations in intestinal metabolome after MBP with recovery to baseline after 14 days. Watanabe et al. [20], in the first study that evaluated the impact of MBP on intestinal microbiota in patients undergoing CRC resection, reported decreased populations of *Bifidobacteria*, *Clostridium coccoides*, *Clostridium leptum*, *Enterobacteriaceae*, and *Lactobacillus* postoperatively, with no change in *Enterococcus* and *Staphylococcus* populations. These findings along with reduced levels of short-chain fatty acids (SCFAs) could result in impairment of the intestinal barrier, thus leading to bacterial translocation and possible infectious complications. The duration of MBP also affects the postoperative microbiota composition after colorectal resection, with reduced numbers of *Bifidobacterium* and *Lactobacillus* in 1-day MBP and increased numbers of *E. coli* and *Staphylococcus* in 3-day MBP, with a significantly lower rate of postoperative infection in the later scheme [21]. In another randomized control trial that investigated the impact of preoperative MBP in the postoperative composition of intestinal microbiota in patients with rectal cancer revealed intestinal dysbiosis with significant reduction in total bacterial abundance, especially in members of *Bacteroides* and *Peptostreptococcus*, and in bacillus/coccus ratio [22].

All the above studies indicate that MBP greatly impacts the diversity and abundance of intestinal microbiota. However, since non-pathogenic bacteria constitute the vast majority of intestinal microbiome, MBP mainly reduces these populations which are also beneficial, such as *Bifidobacteria* and *Lactobacilli*, allowing pathogens to thrive, including *Escherichia coli* (*E. coli)* and *Staphylococcus* [23]. Although there is a non-specific general pattern, the alterations mainly indicated enhanced abundance in *Bacteroidetes* and *Proteobacteria* and decreased numbers of *Lactobacillaceae* after MBP. Notably, these changes are mostly impermanent, and microbial restitution is observed almost two weeks following MBP.

The mechanisms of MBP-related changes in microbiota depend on numerous factors. The osmotic flow in combination with the parallel enhancement of colonic motility act by flushing out luminal and mucosa-associated bacteria, respectively [24,25]. MPB also results in a significant loss of the outer mucus layer, where most microbiota members reside, affecting its production and quality. Moreover, purging leads to a less anaerobic microenvironment through the introduction of oxygen into the colon in addition to increased pH due to the diminishment of SCFAs, which are events that are especially responsible for the increased abundance of *Proteobacteria* [25].

## 3. Perioperative Antibiotic Administration and Intestinal Microbiota

The introduction of antibiotic usage, either oral or systematic, greatly benefited the outcomes of colorectal surgery especially by means of disinfection and control of possible infectious complications. At present, the standard prophylactic antibiotic regimes present broad-spectrum activity; hence, there is no ability to selectively targeting specific pathogens. Antibiotics are also able to alter intestinal microbiota, depending on their pharmacokinetics and antibacterial potential, greatly reducing microbial diversity [26].

Many studies have investigated the effects of antibiotic administration that are commonly used in colorectal surgery on intestinal microbiota. One of the earlier studies by Young et al. [27] showed that 10-day oral administration of amoxicillin–clavulanic acid almost eliminated *Bifidobacterium* spp. by day 4, which did not recover even 20 days later, as opposed to members of *Enterobacteriaceae*. It was also reported that a 5-day oral administration of amoxicillin significantly decreased the diversity and abundance of intestinal microbiota as early as day 1, with its composition being partially recovered to approximately 90% on day 60 [28]. In another study by Dethlefsen et al. [29], a 5-day oral treatment with ciprofloxacin resulted in major taxonomic disruptions that were not fully restored even after 6 months. Notably, this phenomenon is less profound with ciprofloxacin than with amoxicillin–clavulanic acid or clindamycin [30]. The late incomplete recovery of intestinal microbiota composition due to ciprofloxacin actually pertains to a new stable state that differs from its initial form and varies between individuals [31]. Regarding intravenous antibiotics, the administration of β-lactams (ampicillin, cefazolin, and sulbactam) led to a decrease in *Bacteroidetes* and increase in *Firmicutes* after the end of treatment [32]. The administration of β-lactams and fluoroquinolones caused an important reduction (25%) in microbial diversity, increasing the *Bacteroidetes*/*Firmicutes* ratio, although this effect was less profound with levofloxacin/metronidazole and piperacillin/tazobactam [33].

From the aforementioned studies, it becomes evident that perioperative antibiotic therapy, even short-term, could result in deleterious phenomena to the intestinal microbiota persisting for several months. The non-specific antimicrobial action of antibiotics leads to intestinal dysbiosis, possibly favoring the abundance of opportunistic pathogens. These detrimental effects could result in various conditions, such as diarrhea and pseudomembranous colitis [34,35]. Interestingly, it has been reported that prophylactic antibiotic treatment along with its duration are not risk factors for pseudomembranous colitis after CRC surgery [36]. Apart from shifts in intestinal microbiota composition, antibiotics also disrupt the intestinal metabolome, thus impacting host physiology. Antibiotic treatment with concurrent epithelial injury after colorectal surgery could also promote bacterial translocation, hence promoting systemic inflammation and possibly sepsis [37].

Of particular concern is the effect of combined MBP with antibiotics (MOAB) in colorectal surgery, which could induce additional shifts in intestinal microbiota [38]. The current debate is about the selection of MBP with or without oral antibiotics for colorectal procedures. Results from a large prospective analysis (GRECCAR III) showed that the lack of preoperative MOAB was related to an increased risk of infectious complications with no change in AL rate after sphincter-saving rectal resection for rectal cancer [39]. These finding are in line with data from the Cochrane Database of Systematic Reviews, which suggest that MBP is not necessary in colorectal patients, since it does not decrease SSI rates unless MOAB is used [40,41]. Other large retrospective studies have also shown that preoperative MOAB significantly reduces postoperative complications including AL, ileus, and SSI [42,43]. A recent meta-analysis by Rollins et al. [44] confirmed the superiority of MOAB in improving the surgical outcomes after elective colorectal surgery. However, the MOBILE trial demonstrated that MOAB does not decrease overall morbidity or SSI rates after colorectal surgery compared with MBP [45].

In summary, although many studies show beneficial postoperative effects of MOAB in colorectal patients, including CRC cases, there is large discrepancy with other clinical trials. The current guidelines by the American Society for ERAS recommend the routine use of MOAB preoperatively [46]. Thus, it is crucial that ERAS guidelines are reconsidered, emphasizing the necessity for MBP with or without antibiotics in colorectal surgery [47]. Apart from bowel preparation, prophylactic perioperative antibiotic treatment either orally or intravenously is still part of standard surgical care [48], although its importance is still questionable [49].

## 4. Alterations of Intestinal Microbiota Composition Following CRC Surgery

It is well established that the intestinal microbiota interacts with the intestinal epithelium through a compatible, complex, and long-term symbiotic association. Physiologic alterations in the human body caused as a result of surgical stress trigger host-related responses that impact on the intestinal microenvironment, leading to disruption of this delicate ecological equilibrium [50]. Recent animal studies have revealed significant alterations in the composition of the intestinal microbiota after colorectal resection, with reduced abundance in *Bacteroidetes*, especially in *Bacteroidaceae*, and in *Proteobacteria* such as *Enterobacteriaceae* and *Rhodospirillaceae* [51]. In the field of CRC, few studies have investigated the microbial alterations after curative colorectal surgery.

One of the earlier studies by Liu et al. [52] by using bacterial anaerobic cultures and polymerase chain reaction (PCR)-DGGE profiling in fecal samples showed a reduction in *Bifidobacteria* and *Lactobacilli* and an increase in *Enterobacteriaceae*, *Enterococci*, *Pseudomonas*, and *Candida* after CRC surgery. However, these changes did not reach statistical significance, and most members of the fecal microbiota, being obligate anaerobes, cannot be inefficiently cultured through conventional methods. Ohigashi et al. [53] conducted the first study to assess the impact of CRC surgery on the fecal microbiota through 16S rRNA-targeted reverse transcription-quantitative PCR (RT-qPCR) analysis. The results demonstrated a decreased abundance in obligate anaerobic bacteria, including members of *Bacteroides*, *Bifidobacterium*, *Clostridium*, and *Prevotella*, which are crucial for intestinal homeostasis [54], whereas pathogens such as *Enterococcus*, *Pseudomonas*, and *Staphylococcus* were enriched postoperatively. A more recent study by Deng et al. [55] showed decreased numbers of *Firmicutes* and *Bacteroidetes*, contrary to *Proteobacteria* in patients after CRC surgery compared to healthy individuals or CRC patients without surgical treatment via 16S rRNA gene-sequencing of fecal samples. However, the small sampling size (five CRC resections) indicates that these findings may be precarious in drawing any further general conclusions.

Cong et al. [56] reported significantly reduced overall diversity in the fecal microbiota in post-surgery CRC patients compared to healthy individuals and pre-surgery CRC patients. This finding might reflect the further reduction in bacterial diversity as a result of perioperative antibiotic prophylaxis [31]. At the phylum level, the relative abundance of *Proteobacteria* in the post-surgery CRC group was higher than that of the pre-surgery CRC group. Similar results were demonstrated at the genus level regarding *Klebsiella*. In post-surgery CRC patients, *Klebsiella* was correlated with infectious diseases. Of note, this genus is an opportunistic pathogen, causing various infections with high morbidity and mortality [57]. Moreover, in the same group, *Klebsiella* also associated with lymphatic invasion. Intestinal microbiota after CRC surgery showed a looser ecological network than the other groups, pertaining to higher sensitivity to external alterations. It should also be mentioned that all CRC patients suffered from rectal cancer; thus, the results are not fully representative of the composition of colonic microbiota.

A recent study by Kong et al. [58] evaluated the compositional shifts of the intestinal microbiota in fecal samples from 43 CRC patients undergoing radical surgery through 16S rRNA amplicon sequencing preoperatively compared to postoperatively. After CRC surgery, the ratio of *Bacteroidetes*/*Firmicutes* was reduced, which could contribute to intestinal inflammation [59]. The numbers of beneficial obligate anaerobes, including *Bacteroides*, *Bifidobacterium*, *Faecalibacterium*, *Parabacteroides*, and *Prevotella* were also reduced postoperatively. Additionally, radical surgery not only removed the tumoric lesions but also eliminated well-known tumor-associated bacteria including *Enterococcus* and *Fusobacterium*, possibly aiding in recurrence prevention. Furthermore, butyrate-producing bacteria (*Bacillus*, *Bilophila*, *Barnesiella*) were decreased, whereas conditional pathogens including *Escherichia-Shigella*, *Enterobacteriaceae*, and *Streptococcus* were enhanced. These results are contrary to another study that revealed a decrease in *Escherichia-Shigella* and increase in *Enterococcus* and *Parabacteroides* [60]. Thus, these perturbations of intestinal microbiota after CRC surgery could promote adverse inflammatory outcomes in these patients.

## 5. The Role of Intestinal Microbiota in Anastomotic Leakage after CRC Surgery

Immediately after colorectal anastomosis is performed, the tissue-repairing process is initiated and divided into three phases [61]. Firstly, the lag phase occurs consisting of two main events: hemostasis and inflammatory response. In this phase, a fibrin/fibronectin-rich matrix is generated, temporarily bridging the two intestinal ends. The later stages of this phase include the induction of the complementary cascade and the infiltration of the intestinal wound by immune cells. Secondly, the fibroplasia phase occurs marked by fibroblast proliferation and collagen formation. Lastly, in the maturation phase, the collagen is remodeled, and the intestinal epithelium is developed. Ultimately, the healing process leads to the restitution of the intestinal epithelial barrier, and many components of the intestinal microenvironment are involved, including host cells, luminal proliferative components, and microbiota [62]. Indeed, germ-free mice present reduced epithelial regenerative responses and impaired healing of the intestinal barrier as opposed to conventional ones [63]. However, microbiota could either promote or suppress wound healing through competition and/or cooperation between different bacterial species [64].

The resident intestinal microbiota contributes to the physiology of intestinal wound healing and epithelial restitution via various molecular mechanisms. Members of the microbiota are able to interact with intestinal epithelial cells through receptors of the innate immunity, such as toll-like receptors (TLRs), through the recognition of microbiota-related molecular patterns, thus regulating host response [65]. The recognition of commensal members of intestinal microbiota was reported in mice, along with an aggravation of epithelial inflammation and injury in the absence of TLR2, TLR4, and MyD88 [66]. Bacterial metabolites are also beneficial for intestinal epithelial repair, especially SCFAs such as butyrate. Butyrate, a by-product of fiber fermentation, is the main energy source for colonocytes [67], regulating their proliferation, solidifying the intestinal barrier, limiting pathogen growth, and inhibiting proinflammatory cytokines [68]. Animal studies have shown that the oral or rectal administration of butyrate enhanced the bursting wall tension of anastomosis after left or right colectomy [69,70]. Levison et al. [71] demonstrated that SCFAs could hinder the growth of AL-related pathogen *Pseudomonas aeruginosa* (*P. aeruginosa*). Regarding CRC, the production of butyrate by the intestinal microbiota results in significant anti-carcinogenic effects [67]. Other microbiota-derived molecules, such as formyl peptides, bind to associated receptors on the apical surface of colonocytes, namely formyl peptide receptors (FPRs), stimulating the production of reactive oxygen species (ROS) and immune cell chemotaxis [72]. Especially, hydrogen peroxide (H2O2) has been shown to promote various phenomena of anastomotic healing, including vascularization, the induction of epithelial cell proliferation, adhesion, and migration, along with immune stimulation for the prevention of wound infections [73]. This FPR-mediated ROS production is mainly activated by *Lactobacillus* and other butyrate-producing members of intestinal microbiota, and the stimulation of the extracellular signal-regulated kinase/mitogen-activated protein kinase (ERK/MAPK) pathway seems to be the responsible mechanism for the regulation of epithelial recovery [74,75]. Intestinal injury can lead tothe recruitment of polymorphonuclear cells at the wound site and the subsequent respiratory burst results in a hypoxic microenvironment [76]. These events enhance a transient microbial consortium, inducing pro-restitutive signaling cascades and modulating epithelial cell proliferation and migration through the activation of FPR1 and NADPH oxidase (NOX)1 mainly by *Akkermansia muciniphila*. Hence, the above studies suggest that specific members and metabolites of intestinal microbiota are needed for proper wound restoration. Nevertheless, Okada et al. [77] reported the significance of the whole microbiota ecosystem in colonic anastomotic healing, since the anastomotic bursting pressure was higher in conventionalized rats compared to germ-free or monocontaminated rats.

AL constitutes one of the most disastrous postoperative complications following major colorectal procedures, which is associated with increased risk of mortality and morbidity. The incidence of colorectal AL generally ranges from 3% to 20%, with the peak rate being up to 24% after procedures close to the anal verge such as in low colorectal or coloanal anastomoses [78]. Traditionally, AL occurrence was viewed to be a consequence of poor surgical technique-related factors including ischemia, increased suture tension, device deployment, suture type or placement, and stapling method. However, recent vast improvements in surgical technology failed to eradicate AL, worsening its rates in some cases [79]. Recent studies have identified male sex, obesity, diabetes, low preoperative albumin levels, tobacco usage, corticosteroids, and neoadjuvant chemoradiotherapy to be crucial factors for AL development [80,81].

In order to further investigate the pathogenesis of this complication, the intestinal microbiota has been proposed to play a key role in AL development. Furthermore, many of the aforementioned risk factors have been reported to affect the composition of the intestinal microbiota [82]. Many studies have investigated the association between AL and intestinal microbiota. Shogan et al. [83] analyzed the alterations of luminal and mucosa-associated intestinal microbiota in a colectomy rat model through 16S rRNA sequencing analysis, without any preoperative MBP or antibiotic prophylaxis. They revealed a significant increase of the relative abundance of *Escherichia/Shigella* and *Enterococcus* (200- and 500-fold respectively) in the anastomotic tissue. The preponderance of various bacterial virulence-related pathways in the anastomotic tissue was also predicted via further profiling analysis. However, these alterations were observed in tissue-related microbiota contrary to stool microbiota; hence, certain bacteria could express adhesive properties that would lead to invasion in the anastomotic site. Host–pathogen interactions may result in the stimulation of bacterial virulence and subsequent tissue injury [84], detrimentally impacting the restitution of epithelial barrier, thus contributing to AL pathogenesis [85].

In order to investigate the responsible microbiota-related mechanisms of AL, again, Shogan et al. [86] studied a rodent model of low colonic resection and segmental devascularization. Nearly half of the devascularized anastomoses developed AL; however, hypoxic analysis did not reveal ischemia in these segments independently of AL development. They identified that strains of *Enterococcus faecalis (E. faecalis)* colonized leaking tissues, presenting high collagenolytic ability either directly or through the active conversion of matrix metalloproteinase (MMP)-9. The activation of MMP-9 was found to be a result of specific collagenase-encoding genes (*gelE* and *sprE*), thus leading to AL. *Enterococcus* presents high affinity regarding adherence to numerous extracellular matrix (ECM) proteins, such as laminin, fibronectin, and collagen I or IV [87]. Moreover, the anastomotic tissue of patients undergoing colonic resection, following proper intravenous antibiotic prophylaxis, was still colonized with strains of *E. faecalis* and *P. aeruginosa* with collagenolytic/MMP-9 activating properties [86]. Thus, specific bacterial strains could be causal in AL through collagen-degrading potential, which could be recruited through injured tissue signaling, and MMP inhibitors could possibly aid the prevention of AL [86]. The presence of *E. faecalis* in the drainage of patients with colorectal AL indicates its role as a potential screening tool for the early prevention of AL [88].

Adequate vascularization of the intestinal anastomosis is also an important factor in anastomotic healing, since the usage of indocyanine green fluorescence to monitor anastomotic vascularization aid the reduction of AL rates to <2% [89]. Microbiota seems to participate in proper anastomotic vascularization, promoting vascular remodeling [90]. In a study of intestinal ischemia/reperfusion, the diversity of the intestinal microbiota increased significantly at 6 hours post-reperfusion with transient compositional shifts, which was characterized by proliferation of *E. coli*, *Prevotella oralis*, and *Lactobacilli* [91].

Perioperative management also influences AL development in association with intestinal microbiota. MBP results in a thinner fecal consistency, and intestinal contents could possibly leak through the anastomosis. The increased intestinal flow rate impacts the microbiota structure depending on its adherence ability to intestinal mucosa [92]. MBP also leads to mucosal inflammation, modulating the interaction between epithelial cells and ECM, affecting the integrity of the intestinal barrier [93]. Wu et al. [94] have shown that MBP with high-molecular-weight PEG (PEG/Pi), acting as a mucin-like substitute, resulted in AL prevention in rats by suppressing the virulence phenotype of *P. aeruginosa*. Microbiota changes due to antibiotic treatment could also impact intestinal healing [95]. The issue of antibiotic administration and intestinal microbiota in colonic anastomosis was first introduced in 1955 by Cohn and Rives [96]. In this study, dogs undergoing transverse colon resection with pre-anastomotic devascularization received intraluminal infusions daily either with tetracycline or with saline proximal to the anastomotic site. Intraluminal antibiotics successfully prevented AL, reduced mortality, and reversed ischemia [96]. Another study using a similar design in rats showed that oral antibiotics prevented ischemia-related AL as opposed to a high rate (83%) in the control group [97]. Nevertheless, currently, the standard antibiotic prophylaxis for colorectal surgery includes intravenous broad-spectrum antibiotics. The oral antibiotics were reintroduced in the context of MOAB, with studies demonstrating a postoperative reduction of AL following colectomy to almost 50% [42]. A more recent clinical trial further confirmed that the usage of MOAB significantly decreases AL rates in patients after CRC surgery [9]. Opioids used in perioperative analgesia, such as morphine, have been demonstrated to promote the adherence of collagenolytic *E. faecalis* to the anastomotic site, leading to AL in rats [98].

Another intervention of particular interest is radiotherapy, which is mainly administered prior to CRC surgery as part of neoadjuvant treatment, especially in advanced rectal cancer. Radiotherapy has been shown to contribute to AL, increasing its rate by threefold [99]. Preoperative radiation has also been reported to induce hypoperfusion effects in the intestinal environment [100], leading to a reduction of beneficial obligate anaerobic bacteria including *Bacteroides* and *Clostridia*, while promoting *Enterobacteriaceae* and *Lactobacillus* [101]. The disturbance of the commensal microbiota in the irradiated intestinal mucosa is of major importance, since bacterial TLRs are crucial for the survival of the intestinal epithelium from the radiation-related apoptosis through the activation of nuclear factor kappa B (NF-κΒ) signaling [102]. Furthermore, the resultant mucositis, vascular injury, and epithelial apoptosis could enable virulent phenotypes of intestinal microbiota [103]. To further investigate the radiation effect on intestinal microbiota in association with AL, Olivas et al. [104] exposed rats to preoperative radiation followed by low anterior resection with primary anastomosis and postoperative intestinal inoculation with *P. aeruginosa*, which is a well-known colonizer of irradiated intestine [105]. AL was only developed in rats with both intestinal inoculation by *P. aeruginosa* and radiation. The phenotype analysis of *P. aeruginosa* strains from AL rats revealed in vivo single nucleotide polymorphism in the *mexT* gene, resulting in enhanced collagenolysis, cytotoxicity, and invasion. Thus, intestinal pathogens could in vivo shift into virulent phenotypes through the emission of various factors by the irradiated intestinal tissue causing AL. This study further supports the importance of host–pathogen interactions in the pathogenesis of AL.

The available literature regarding the relationship between the intestinal microbiota and AL in human patients with CRC is very limited. A recent study by van Praagh et al. [106], based on the results from a previous pilot study [68], explored this association in patients that were included in a trial, aiming to assess the efficacy of this bioresorbable sheath (C-seal) in preventing colorectal AL [107]. The mucosa-associated microbiota was sampled from the stapled colorectal “donut” and analyzed through 16S MiSeq sequencing. The results showed that in non-C-seal patients, AL was strongly related to reduced microbial diversity and an increased abundance of *Bacteroidaceae* and *Lachnospiraceae* compared to matched patients without AL [106]. Contrarily, AL was not associated with intestinal microbiota in C-seal patients, which presented a higher AL rate than the non-C-seal group (27% vs. 17%). Although C-seal placement did not reduce AL occurrence, it obliterated the association between intestinal microbiota and AL. Further analysis indicated that an intestinal microbiota composition consisting of ≥60% *Bacteroidaceae* and *Lachnospiraceae* is predictive for AL. Of note, these bacterial families are crucial to the homeostasis of the colonic epithelial cells, with the *Lachnospiraceae* family including many butyrate-producing members, such as *Eubacterium rectale* and *Faecalibacterium prausnitzii* [108]. It should also be mentioned that from the total of 123 patients, only 117 underwent colorectal resection for CRC. A more recent study by Palmisano et al. [109] compared the preoperative microbiota composition in patients eligible for CRC surgery before any perioperative intervention. Patients who developed AL demonstrated an increase in dysbiosis-related bacteria *Acinetobacter lwoffi* and *Hafnia alvei*. Interestingly, *Acinetobacter lwoffi* is a member of normal oropharyngeal microbiota in 25% of individuals [110], possibly supporting the hypothesis of orally-driven intestinal dysbiosis in CRC [111]. Patients with AL also showed low abundances of protective bacteria *Barnesiella intestinihominis* and *Faecalibacterium prausnitzii* [109]. Thus, determination of the preoperative composition of colonic microbiota could serve as a potential screening tool for AL in CRC patients.

**Figure 1:**
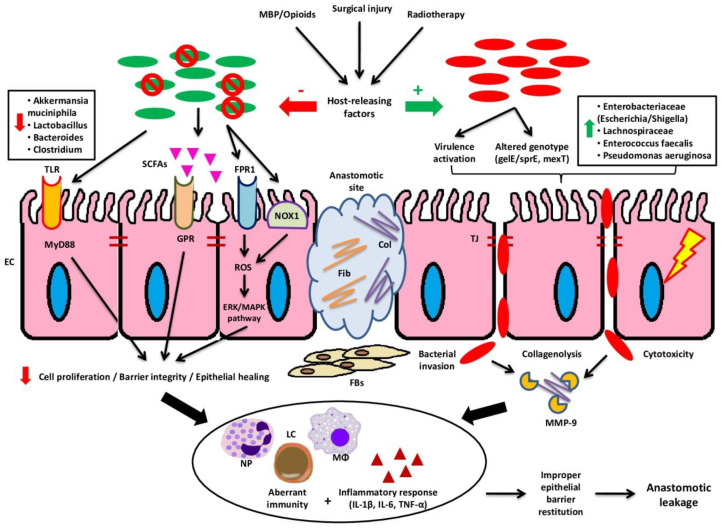
Events after colorectal surgery leading to AL. Surgical injury following colorectal resection in combination with perioperative interventions (MBP, opioids, and radiotherapy) trigger the release of various host-derived factors. These factors interfere with the intestinal microbiota at the anastomotic site causing shifts. Physiologically, microbiota promotes colonic cell proliferation and epithelial healing and enhances the integrity of the intestinal barrier, mainly through the stimulation of TLRs, release of SCFAs (e.g. butyrate), and interaction with FPR1 and NOX1 causing the release of ROS and upregulation of the ERK/MAPK pathway. However, the deleterious effects of the aforementioned factors result in reduction of beneficial members of microbiota, thus suppressing normal wound healing. Moreover, several pathogens are increased demonstrating virulence activation and altered genotype. These effects lead to bacterial invasion through failure of epithelial tight junctions, collagenolysis through the activation of MMP-9, and cytotoxicity. The transformation of the intestinal microbiota into a “pathobiome” results in aberrant immunity and inflammatory response, which impair the proper epithelial restitution, eventually leading to the pathogenesis of AL. AL: anastomotic leakage; Col: collagen; EC: epithelial cell; ERK: extracellular signal-regulated kinase; FBs: fibroblasts; Fib: fibrin/fibronectin; FPR: formyl peptide receptor; GPR: G-protein coupled receptor; IL: interleukin; LC: lymphocyte; MAPK: mitogen-activated protein kinase; MBP: mechanical bowel preparation; MMP: matrix metalloproteinase; MΦ: macrophage; NOX: NADPH oxidase; NP: neutrophil; ROS: reactive oxygen species; SCFAs: short-chain fatty acids; TLR: toll-like receptors; TNF: tumor necrosis factor.

The responsible mechanisms for the pathogenesis of AL in colorectal surgery are summarized in Figure 1.

## 6. Surgical Site Infections in Relation to Intestinal Microbiota

Infectious complications following colorectal surgery still have high rates, resulting in increased cost and readmission [112]. Of particular interest in colorectal surgery are SSIs, and there is evidence to support that intestinal microbiota may be involved in the pathogenesis of these complications.

SSI is a common and unfavorable complication, occurring in 3–26% in patients undergoing colorectal surgery [113]. SSI actually pertains to an infection occurring within 30 days postoperatively and being classified as either incisional (superficial or deep) or organ/space SSI. The most common pathogen is *Staphylococcus aureus (S. aureus)*, being causal in 20% of SSIs [114].

Although direct intraoperative contamination and/or the spillage of intestinal contents are obvious risk factors for SSIs, wound cultures are often poor predictors of postoperative SSIs [115]. Furthermore, SSIs still occur after targeted systemic antibiotic treatment or in the absence of bowel manipulation and intraoperative contamination/spillage, being especially colonized by methicillin-resistant *S. aureus* (MRSA) [116]. Hence, more mechanisms may be involved in SSI pathogenesis. According to a proposed mechanism of microbiota-mediated SSIs, the “Trojan-horse hypothesis”, low-abundant intestinal pathogens, such as MRSA, could translocate inside a macrophage or neutrophil to remote wound sites, resulting in clinical infection [117]. A recent study confirmed that intestinal colonization via oral MRSA gavage administration in combination with antibiotic treatment led to distant wound infection with abscess development in mice through the intra-neutrophilic translocation of MRSA [118]. Similar findings have been demonstrated in distant SSIs caused by *E. faecalis* [112]. It has also been reported that intestinal microbiota are able to promote surgical incision healing through the recruitment of T-cells, which is mediated through the release of oxytocin after stimulation of the vagus nerve as a result of the fermentation of lactic acid [119]. The disruption of intestinal microbiota after surgical stress could possibly impair this mechanism, leading to improper wound healing, contamination from members of skin microbiota (such as *S. aureus*), and subsequent SSI.

One of the primary reasons for the introduction of preoperative MOAB in the clinical practice of colorectal surgery was the need for the proper intestinal decontamination from pathogens that could cause postoperative SSI. Clarke et al. [120] revealed a significant decrease in SSI rate after elective colorectal procedures from 35% in the MBP group to 9% in the MOAB group. Similar findings were demonstrated in more recent studies, favoring the use of preoperative MOAB in SSI prevention [121,122]. However, other trials showed that MOAB was not efficient in reducing SSI rates after colorectal surgery compared with MBP [45]. This fact combined with the current lack of high-quality Level 1 evidence for MOAB usage in the prevention of postoperative infectious complications in colorectal procedures still points to MOAB application as a subject of ongoing debate [123].

Studies investigating SSI incidence and risk factors in patients after CRC surgery have also been conducted. Huh et al. [124] assessed the oncological outcome of SSI in 3675 CRC patients following curative resection. The overall SSI rate was found to be 9.6%, of which 5.5% were incisional SSIs and 4.1% were organ/space SSIs. The rates of SSIs were almost two-fold in rectal cancer compared with colon cancer cases (14% vs. 7%). Notably, SSI predicted disease-free survival, whereas patient characteristics, tumor location, and perioperative conditions were independent predictors of SSI. A more recent multicenter study in 5729 CRC patients eligible for curative resection confirmed the increased incidence of all types of SSI in rectal cancer as opposed to colon cancer [125]. The most common causal pathogens of SSIs were also identified, being *E. coli* and *P. aeruginosa* in colon cancer, and *E. coli* and *Enterococcus* spp. in rectal cancer. However, both studies showed a lack of analysis of the intestinal microbiota and correlation with these results. Ohigashi et al. [53] is currently the only study that associated fecal microbiota with SSI-related bacteria in CRC patients after surgery. SSI occurred in six out of 81 patients, and the causative bacteria were identified to be *S. aureus*, *P. aeruginosa*, and *Enterococcus* spp., which were also enriched in the analysis of fecal samples after CRC surgery. These results might be supportive of the “Trojan-horse hypothesis” and enhanced bacterial virulence as possible mechanisms of infectious complications in patients after CRC surgery.

## 7. Intestinal Microbiota and Long-Term Outcomes Following CRC Surgery

Apart from the postoperative complications, studies have also investigated the intestinal microbiota in association with long-term outcomes following CRC surgery.

The most commonly studied bacterium in CRC patients in relation to oncological outcomes is *Fusobacterium nucleatum (F. nucleatum)*. In particular, *F. nucleatum* has been indicated to be involved in the pathogenesis of CRC, especially in adenoma-CRC progression, since increased numbers of this bacterium were detected in colorectal adenomas compared to healthy tissue [126]. *F. nucleatum* could promote CRC tumorigenesis through numerous mechanisms, mainly through the binding of its adhesion protein FadA to E-cadherin and activation of the β-catenin pathway [127]. The hypothesis of *F. nucleatum*–adenoma interaction has been validated by Flanagan et al. [128], demonstrating the correlation of stages of adenoma–CRC progression with an increased abundance of *F. nucleatum*. The majority of relevant studies have significantly correlated higher levels of *F. nucleatum* with unfavorable outcomes, including reduced overall survival, cancer-specific survival, disease-free survival, or median survival, and increased cancer-specific mortality [128,129]. Furthermore, the presence of this bacterium has been associated with larger tumor size, advanced tumor stage, and increased tumor invasion or lymph node metastasis [129,130]. The worse prognosis and metastatic potential of CRC can be predicted through high levels of interleukin (IL)-17, and increased cancer-related mortality is associated with an inhibition of CD3+ and CD4+ T-cells [131]. Interestingly, *F. nucleatum* has been indicated to be a causal factor of these alterations [126,132], suggesting the involvement of host-immunity/microbiota interaction in modulating the outcomes in CRC patients.

Wei et al. [133] have also reported that similar to *F. nucleatum*, *Bacteroides fragilis (B. fragilis)* is correlated with reduced disease-free and overall survival. Especially enterotoxigenic *B. fragilis* (ETBF) has been found to be related to CRC progression, since a higher abundance of ETBF has been detected in tubular adenomas or low-grade colorectal dysplasia [134]. Advanced stages of CRC were also related to increased levels of ETBF, and this bacterium can stimulate cytokine-release through the NF-κΒ pathway [135].

Another bacterial genus of particular interest concerning CRC outcomes is *Bifidobacterium*, with its abundance having been reduced in CRC patients compared with healthy individuals [136]. *Bifidobacteria* possibly acts as a protective probiotic in CRC, acting by enhancing intestinal epithelial homeostasis [137]. Kosumi et al. [138] assessed the prognostic potential of *Bifidobacterium* in a cohort study of 1313 CRC patients. Contrary to the above findings, they reported the detection of *Bifidobacterium* in 30% of tumors with no statistically significant association between its abundance and survival outcomes or molecular and clinicopatholigical features of CRC. Moreover, *Bifidobacterium* numbers were related to the presence of signet-ring cells. The lack of association between *Bifidobacterium* and CRC outcomes could be due to the production of acetate or lactate by this bacterium stimulating tumor growth or complex interplay with other bacterial species.

Flemer et al. [139] analyzed and compared the tissue-related co-abundance groups (CAGs) of various bacteria, and they reported that specific CAGs (*Bacteroidetes*, *Prevotella*, and pathogen) were correlated with longer survival in CRC patients. These results were contrary to previous findings which proposed that high numbers of *Prevotella* and pathogen CAGs were linked to the stimulation of inflammatory response and reduced survival in CRC patients [140]; thus, further studies are needed to evaluate these data. Other bacterial species, including *Faecalibacterium prausnitzii*, have also been associated with favorable survival outcomes in CRC [133].

Recurrence incidence following curative CRC surgery is reported to be as high as 40%, mainly within 3 years after operation, with local recurrence occurring in 1–23% of CRC cases [141]. Indications of the recovery of the intestinal microbiota composition similar to healthy individuals after surgery for CRC or colorectal adenoma point toward the role of the microbiota as a potential biomarker for tumor recurrence [142]. High levels of *F. nucleatum* were correlated with CRC recurrence, which was probably due to chemotherapy resistance induced by stimulation of the autophagy pathway [143]. A more recent study demonstrated that the intestinal microbiota of newly-developed adenoma patients was significantly different from tumor-free post-surgery CRC patients, having been similar to that of pre-surgery CRC patients [144]. The correlation of these results with CRC prognosis indicated the potential predictive and preventive value of microbiota in recurrence after CRC surgery.

Higher rates of local recurrence have been reported in perianastomotic extraluminal sites, and AL has been related to reduced disease- and recurrence-free survival [145]. Four different mechanisms were hypothesized for explaining these phenomena in AL: (a) implantation of exfoliated tumoric cells to the anastomotic tissue [146], (b) metachronous tumorigenesis [147], (c) tumorigenesis mediated by persistent inflammation [148], and (d) attraction of circulating CRC cells to the anastomotic tissue [149]. These mechanisms have been related to intestinal microbiota changes due to surgical stress and AL [150]. The rise of the pathobiome, after suppression of the commensal microbiome [151], and subsequent aberration of immunity [152] could result in enhanced inflammation and implantation of tumoric cells at the anastomotic tissue, although the precise mechanisms are yet to be elucidated. This pathobiome especially in AL mainly consists of collagenolytic bacteria, such as *E. faecalis* and *P. aeruginosa*, which could promote inflammation through the activation of MMP-9. High MMP-9 levels have been demonstrated to be indicative of tumor invasion and worse outcomes in CRC patients [153]. Moreover, these bacteria could stimulate the expression of the invasive mesenchymal-like phenotype in mouse colonic epithelium via an interaction with local macrophages [154].

In recent study, Lin et al. [155] were the first to evaluate the long-term metabolic alterations and microbial composition in CRC patients after curative surgery. Metabolic syndrome was more prevalent among CRC patients after right hemicolectomy (RH) but not after lower anterior resection (LAR) compared to controls over a follow-up period of 5 years. Furthermore, the RH group demonstrated intestinal dysbiosis through lower bacterial richness and diversity as opposed to the LAR group. The RH group also showed microbiota alterations postoperatively with a reduction of *Firmicures*/*Bacteroidetes* ratio, with a higher level of *Fusobacterium* and lower numbers of butyrate-producing *Faecalibacterium prausnitzii* and *Roseburia*. These results are supportive of the above findings; however, correlation of these microbiota alterations with survival outcomes was not performed.

## 8. Conclusions, Recommendations, and Future Perspectives

The majority of studies regarding MBP and microbiota, apart from being retrospective, are multifactorial since postoperative microbiota composition is further influenced by surgical stress, other perioperative management, and systemic conditions related to the patient. Perioperative antibiotic administration could possibly disrupt intestinal microbiota, resulting in enhanced susceptibility to opportunistic infections, impaired host immunity or metabolism, and an accumulation of antibiotic resistances, which persist long after the end of treatment [95]. Nevertheless, it should be stressed that these conclusions have been drawn from studies that investigated the antibiotic effects on intestinal microbiota in more long-term administration than perioperatively (5, 7, or 10 days) in non-surgical diseases. Studies regarding antibiotic prophylaxis for colorectal surgery rely on culture-based methods [156], questioning their validation compared to modern genomic analysis. Furthermore, longer antibiotic therapy could magnify the effects on microbiota, aiding their interpretation. Probably the most important factor is the “baseline microbiota” of each patient, which could be already altered by the disease itself. In the case of CRC, this is particularly true, since chronic inflammatory response in CRC further impacts on the diversity of the “baseline microbiota” [157], yielding questions of the extent of disease-related observed changes after perioperative interventions, and there are limited studies stressing this issue. The individualized composition of intestinal microbiota may be responsible for the contradictory results from MOAB and MBP studies. Furthermore, since most of these trials do not account for the intestinal microbiota, the responsible microbiota-driven mechanisms that decrease complication rates remain unknown. Nevertheless, MBP should be viewed by colorectal surgeons in terms of convenience, postoperative complications, available guidelines (ERAS), and their self-experience. The selection of proper bowel preparation should be personalized with a fixed selection of antibiotics targeting specific pathogens that prevent surgical complications and support the functions of beneficial microbiota members [158]. Mapping of the intestinal microbiota through high-resolution genetic analysis could be used for a more complete understanding of the interrelation between surgical outcomes and perioperative management in CRC patients. Moreover, more future studies should focus on metabolomics, enabling the investigation of impaired metabolic pathways and altered metabolites, thus further defining the impact of perioperative management and surgical stress on the global metabolic status regarding host–microbiota interactions.

The above studies indicated a significant alteration of intestinal microbiota in response to CRC surgery. Table 1 describes these postoperative changes along with respective alterations in CRC patients compared to healthy individuals [159,160,161,162,163]. Nevertheless, it should be taken into consideration that postoperative microbial changes are not solely depended on surgical stress but also on perioperative interventions.

**Figure 2:**
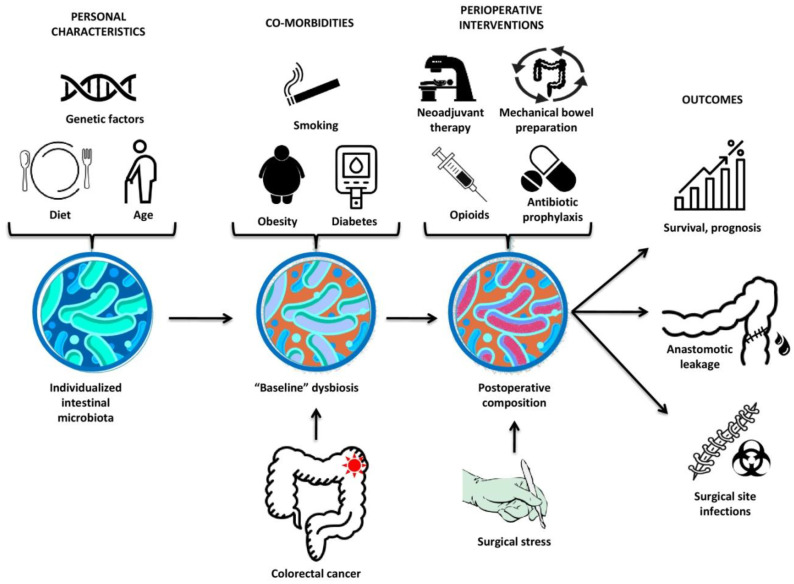
Factors influencing intestinal microbiota composition and outcomes after CRC surgery. Each individual has a distinct intestinal microbiota composition, which is mainly shaped by their age, genetic factors, and dietary preferences. Colorectal cancer, as the primary disease along with common co-morbidities including excessive tobacco usage, obesity, and diabetes further shifts the intestinal microbiota leading to a “baseline” dysbiotic state. The surgical stress combined with various perioperative interventions (neoadjuvant therapy, mechanical bowel preparation, opioids, and antibiotic prophylaxis) result in an altered postoperative microbial composition that ultimately determines the rates of postoperative complications (anastomotic leakage, surgical site infections) and long-term outcomes (survival, prognosis).

All these factors combined could impair the host–microbiota balance, resulting in inflammatory responses and the profusion of pathogens [164], ultimately determining the surgical outcomes (Figure 2). There are also some limitations to these studies. Firstly, the sampling size was relatively small; hence, larger cohort studies would validate these results. Secondly, all of these studies used fecal samples for intestinal microbiota analysis as opposed to tissue samples. The latter samples may represent the local (tumor-adjacent) microbiota but are more relevant to monitor the bacterial shifts that participate in the adenoma–CRC sequence. Lastly, some of these studies are cross-sectional, and more accurate comparative results require the determination of pre- and postoperative microbiota in the same patient.

Regarding surgical complications, the available literature suggests that the intestinal microbiota plays an important role in anastomotic healing and pathogenesis of AL. All this knowledge mainly derives from animal studies. More human trials are needed to evaluate these effects in CRC patients. Specific collagenolytic pathogens, such as *E. faecalis* and *P. aeruginosa*, have been involved in AL pathophysiology. However, their presence is not solely sufficient in developing AL, since other conditions are also needed. These include microbiota perturbations with reduced beneficial bacteria, inflammatory response from anastomotic site, and enhanced microbial virulence, eventually resulting in intestinal dysbiosis [112]. Again, various intra- and perioperative factors could affect intestinal microbiota toward these conditions. In particular, the worse oncological outcomes in CRC patients with AL indicate the need for investigation of the above interactions [80,81]. Analysis of the intestinal microbiota through next-generation technology over the course of anastomotic healing may elucidate which aspects of the microbiota should be obliterated or sustained for the adequate support of the healing process. Due to the fact that perioperative management is still not unified between CRC patients, the characterization of pre- and postoperative microbiota composition is mandatory in shaping guidelines for clinical practice. The aforementioned findings also suggest the existence of a relationship between the intestinal microbiota and postoperative infectious complications, especially SSIs. Nevertheless, the responsible mechanisms for this interaction remain elusive. *P. aeruginosa* and *S. aureus* are the most commonly responsible bacteria for these effects. Whether the alterations in intestinal microbiota are causal or act as indicators of such complications is still ambiguous. Specific bacteria-targeted preoperative preparation could be useful in preventing the emergence of infectious pathogens. In addition, probiotics, prebiotics, or synbiotics could be a valuable adjuvant treatment in CRC surgery. Administration of a four-probiotic regimen (*Lactobacillus acidophilus*, *Lactobacillus plantarum*, *Bifidobacterium lactis,* and *Saccharomyces boulardii*) significantly reduced all postoperative complications following CRC surgery, including SSI and AL [165]. However, in another study, the preoperative administration of prebiotics or synbiotics did not affect complication rates after CRC surgery [166]. Regarding fecal microbiota transplantation (FMT), although current evidence in CRC patients is limited to the modulation of immunotherapy efficacy [167], it has recently gained acceptability for interventional trials in patients after low anterior resection for rectal cancer, which might be promising in restoring the bacterial diversity postoperatively [168].

The aforementioned studies indicate intestinal microbiota as a potential diagnostic or prognostic non-invasive biomarker in CRC patients. More specifically, *F. nucleatum* and *B. fragilis* were associated with worse survival outcomes, whereas *Bifidobacteria* seem to be beneficial in preventing CRC carcinogenesis. The early detection of *F. nucleatum* preoperatively could guide future strategic alterations in CRC management, possibly through the adjustment of adjuvant chemotherapy, close monitoring for recurrence, lymph-node debulking, and more radical resection. Notably, since *F. nucleatum* is resident species in the oral cavity, an assessment of oral microbiota could predict long-term outcomes in CRC [111]. Nevertheless, these results should be cautiously interpreted, since other confounding variables that determine surgical outcomes are partially considered by the majority of these studies.

In summary, this review indicates an important effect of perioperative interventions, mainly MBP and antibiotic prophylaxis on the intestinal microbiota. Postoperative microbial composition is significantly altered after CRC surgery. Moreover, the intestinal microbiota seems to be associated with the development of postoperative anastomotic and infectious complications and the long-term oncological outcomes. Nevertheless, the available evidence is still limited, and more studies should be conducted, especially for CRC surgery.

## Figures and Tables

**Table 1:** Intestinal microbiota composition in colorectal cancer (CRC) patients and after CRC surgery.

Phylum	Genus	CRC vs. Healthy	Post-Op CRC vs. Pre-Op CRC	References
FIRMICUTES				
	*Bacillus*	n/s	↓	[58]
	*Clostridium*	↓	↓	[53,159]
	*Enterococcus*	↑	↑↓	[52,53,58,60,160]
	*Faecalibacterium*	↑	↓	[58,155,159]
	*Lactobacillus*	↓	↓	[52,161]
	*Roseburia*	↓	↓	[155,162]
	*Staphylococcus*	↑	↑	[53,163]
	*Streptococcus*	↑	↑	[58,162]
BACTEROIDETES				
	*Bacteroides*	↓	↓	[53,58,162]
	*Barnesiella*	n/s	↓	[58]
	*Parabacteroides*	n/s	↑↓	[58,60]
	*Prevotella*	↑	↓	[53,58,159]
ACTINOBACTERIA				
	*Bifidobacterium*	↓	↓	[52,53,58,161]
PROTEOBACTERIA				
	*Bilophila*	n/s	↓	[58]
	*Escherichia*	↑	↑↓	[58,60,162]
	*Klebsiella*	↑	↑	[56,162]
	*Pseudomonas*	n/s	↑	[52,53]
	*Shigella*	↑	↑↓	[58,60,162]
FUSOBACTERIA				
	*Fusobacterium*	↑	↑↓	[58,155,159]

↑: higher abundance; ↓: lower abundance; ↑↓: higher or lower abundance; n/s: non-specific; CRC: colorectal cancer; post-op: postoperative; pre-op: preoperative; vs.: versus.
